# Hyperin protects against LPS-induced acute kidney injury by inhibiting TLR4 and NLRP3 signaling pathways

**DOI:** 10.18632/oncotarget.13010

**Published:** 2016-11-01

**Authors:** Gong Chunzhi, Li Zunfeng, Qin Chengwei, Bu Xiangmei, Yu Jingui

**Affiliations:** ^1^ Department of Anesthesiology, Qilu Hospital, Shandong University, Jinan, 250012, China; ^2^ Department of Anesthesiology, Affiliated Hospital of Binzhou Medical University, Binzhou, 256603, China

**Keywords:** hyperin, LPS, kidney injury, TLR4, NLRP3

## Abstract

Hyperin is a flavonoid compound derived from *Ericaceae*, *Guttifera*, and *Celastraceae* that has been shown to have various biological effects, such as anti-inflammatory and anti-oxidant effects. However, there is no evidence to show the protective effects of hyperin on lipopolysaccharide (LPS)-induced acute kidney injury (AKI). Therefore, we investigated the protective effects and mechanism of hyperin on LPS-induced AKI in mice. The levels of TNF-α, IL-6, and IL-1β were tested by ELISA. The effects of hyperin on blood urea nitrogen (BUN) and serum creatinine were also detected. In addition, the expression of TLR4, NF-κB, and NLRP3 were detected by western blot analysis. The results showed that hyperin significantly inhibited LPS-induced TNF-α, IL-6, and IL-1β production. The levels of BUN and creatinine were also suppressed by hyperin. Furthermore, LPS-induced TLR4 expression and NF-κB activation were also inhibited by hyperin. In addition, treatment of hyperin dose-dependently inhibited LPS-induced NLRP3 signaling pathway. In conclusion, the results showed that hyperin inhibited LPS-induced inflammatory response by inhibiting TLR4 and NLRP3 signaling pathways. Hyperin has potential application prospects in the treatment of sepsis-induced AKI.

## INTRODUCTION

Acute kidney injury (AKI), as a serious disease, suffered about one in five patients in emergency cases [1]. In hospitalized patients, AKI becomes a common and dangerous factor for death progressively [2, 3]. AKI can be caused by variety factors, such as pharmacologic toxins and sepsis [4, 5]. LPS, the outer membrane component of gram-negative bacteria, has been identified as the major factor that leads to AKI [6]. In the mice model of LPS-induced AKI, LPS significantly induces the release of inflammatory cytokines which promote kidney disease [7]. Studies showed that inflammatory cytokines TNF-α, IL-6,and IL-1β played critical roles in the pathologicprocess of kidney injury [8]. And inhibition of these inflammatory cytokines could attenuate the injury of kidney tissues. Thus, early anti-inflammatory therapy can improve renal function.

Hyperin, a flavonoid compound found in *Ericaceae*, *Guttifera,* and *Celastraceae*, has been reported to have anti-inflammatory effects [9]. Hyperin has been reported to inhibit LPS-induced nitrite production in rat peritoneal macrophages [10]. Hyperin also inhibited LPS-induced acute liver injury in mice [11]. Furthermore, hyperin has been found to protect the heart against ischemia and reperfusion lesions [12]. However, whether hyperin has protective effects against LPS-induced AKI remains unclear. Therefore, in the present study, we investigated the protective effects and mechanism of hyperin on LPS-induced AKI in mice.

## RESULTS

### Effects of hyperin on LPS-induced kidney histopathologic changes

**Figure 1:**
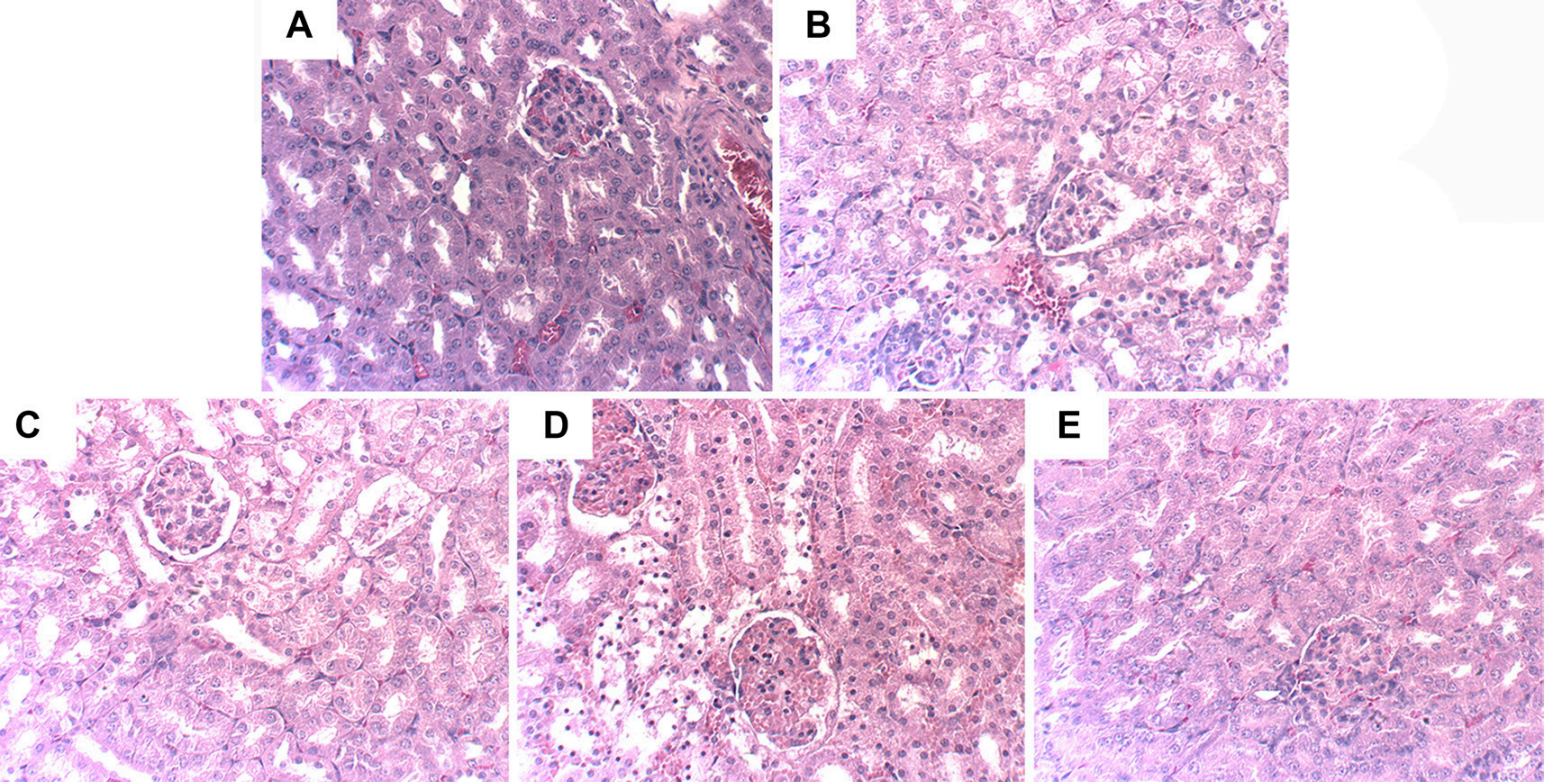
Effects of hyperin on histopathological changes in kidney tissues in LPS-induced AKI mice Hhyperin (25, 50, 100 mg/kg) were given intraperitoneally (i.p.) 1 h after LPS treatment. 24 h after LPS challenge, Kidney tissues from each experimental group were processed for histological evaluation. Representative histological changes of kidney obtained from mice of different groups. (**A**) Control group, (**B**) LPS group, (**C**) LPS+ hyperin (25 mg/kg) group, (**D**) LPS+ hyperin (50 mg/kg) group, (**E**) LPS + hyperin (100 mg/kg) group (Hematoxylin and eosin staining, magnification 200×).

To investigate the protective effects of hyperin, we applied H&E staining to detect the histological changes in renal tissues comparing normal tissues to injured tissues. As shown in Figure 1A, the renal tissues exhibited normal morphology in control group. The renal tissues of LPS group showed severe injury on renal tissues, including tubular cells sloughing, loss of brush, and apoptosis of nephron (Figure 1B). However, the injury was significantly ameliorated by hyperin (Figure 1C, 1D, 1E).

### Hyperin relieves the dysfunction of kidney function of AKI

**Figure 2:**
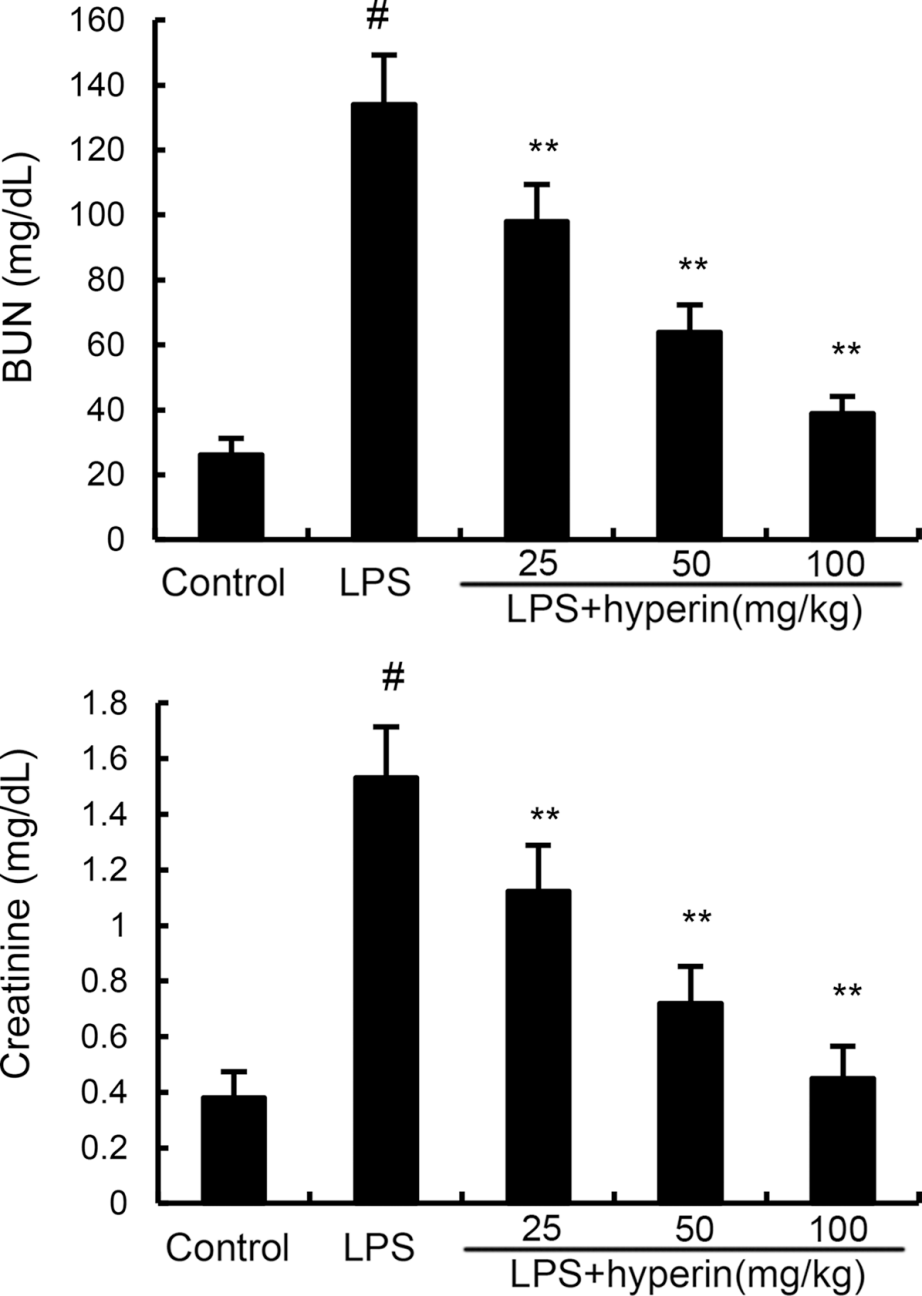
Effects of hyperin on BUN and creatinine levels in serum The values presented are the mean ± SEM (*n* = 12 in each group). ^#^*p* < 0.01 vs. control group, *p < 0.05, **p < 0.01 vs. LPS group.

The levels of BUN and creatinine were measured to assess renal function. As shown in Figure 2, compared with the control group, BUN and creatinine levels were found to be dramatically increased in the LPS group. However, the levels of BUN and creatinine induced by LPS were dose-dependently inhibited by hyperin (25, 50, 100 mg/kg).

### Hyperin inhibits the expression levels of cytokines

**Figure 3:**
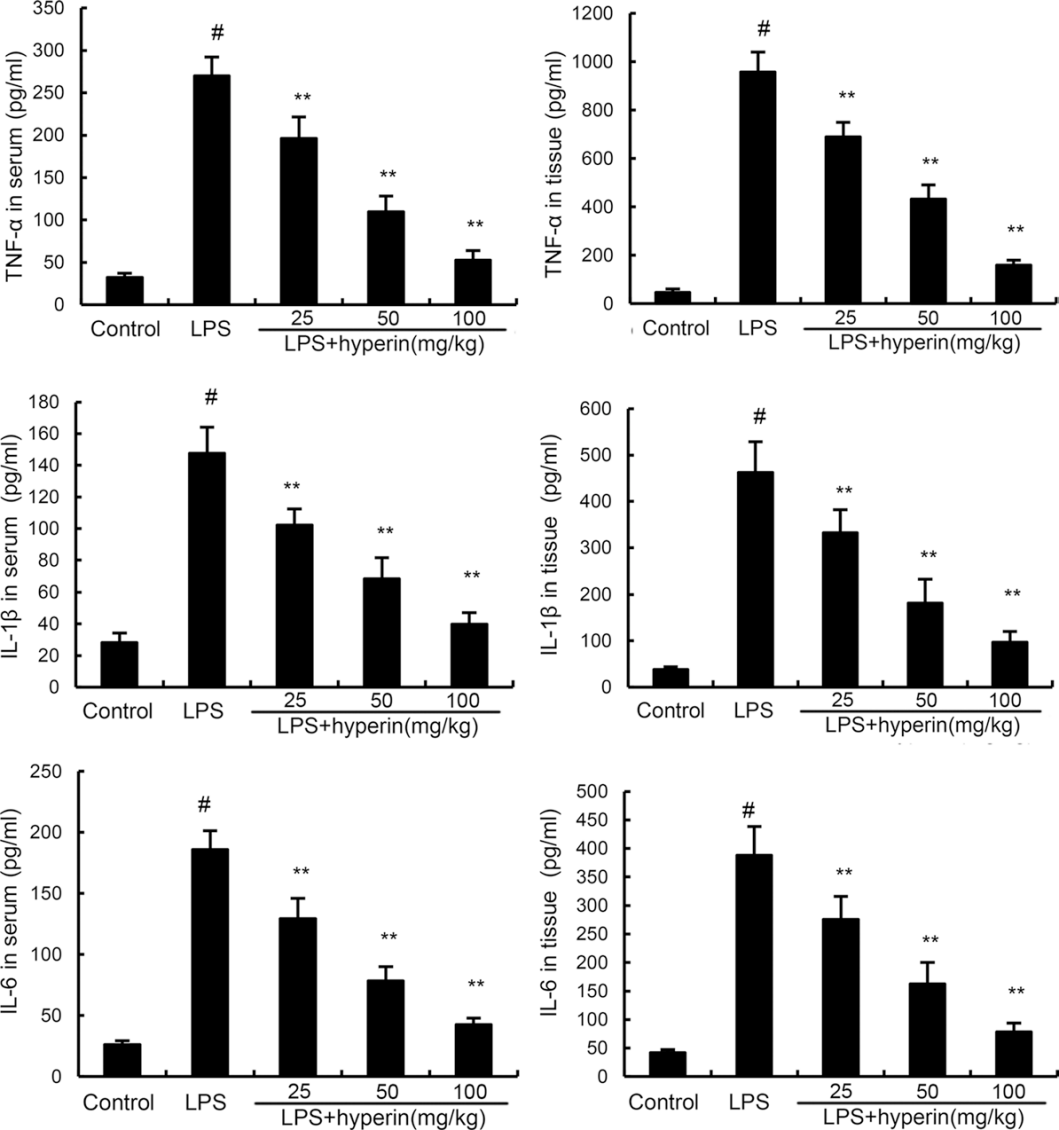
Effects of hyperin on LPS-induced TNF-α, IL-6 and IL-1β in serum and kidney tissues The values presented are mean ± SEM (*n* = 12 in each group). ^#^*p* < 0.01 vs. control group, ^*^p < 0.05, ^**^p < 0.01 vs. LPS group.

To investigate the anti-inflammatory effects of hyperin, the levels of inflammatory cytokines TNF-α, IL-6, and IL-1β production were detected by ELISA. As shown in Figure 3, compared with the control group, the levels of TNF-α, IL-6, and IL-1β were found to be dramatically increased in the LPS group. However, treatment of hyperin significantly inhibited LPS-induced TNF-α, IL-6, and IL-1β production.

### Hyperin inhibits LPS-induced TLR4 expression and NF-κB activation

**Figure 4:**
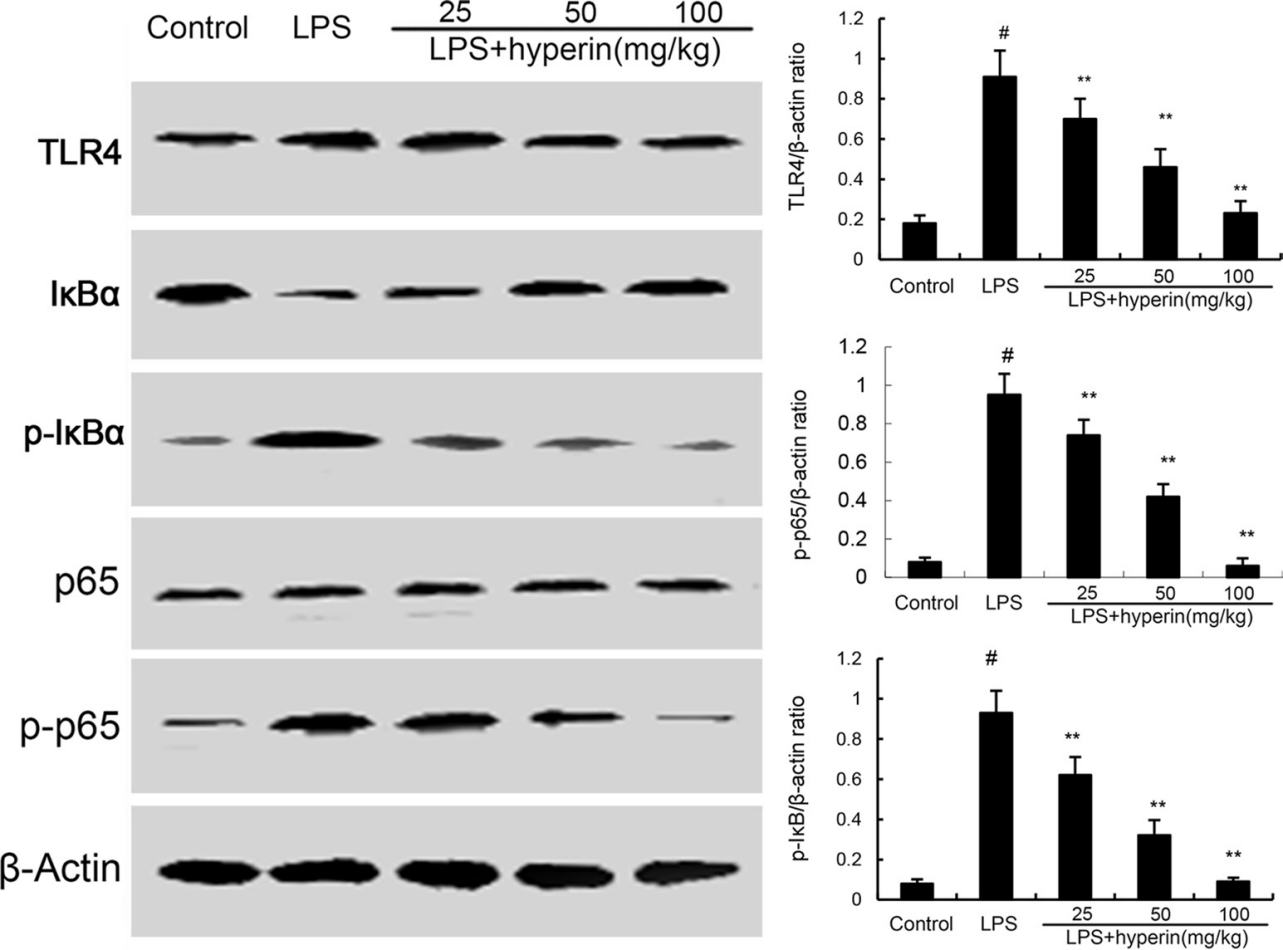
Hyperin inhibits LPS-induced TLR4 expression and NF-κB activation The values presented are the means ± SEM (*n* = 12 in each group). ^#^*p* < 0.01 vs. control group, ^*^p < 0.05 and ^**^p < 0.01 vs. LPS group.

TLR4 plays an important role in LPS-induced acute kidney injury. To investigate the anti-inflammatory mechanism of hyperin, we investigated the effects of hyperin on TLR4 signaling pathway. As shown in Figure 4, LPS challenge significantly up-regulated the expression of TLR4 and activated NF-κB. However, hyperin significantly inhibited LPS-induced TLR4 expression. Furthermore, treatment of hyperin dose-dependently inhibited phosphorylation of NF-κB and IκBα.

### Hyperin inhibits LPS-induced NLRP3 signaling pathway

**Figure 5:**
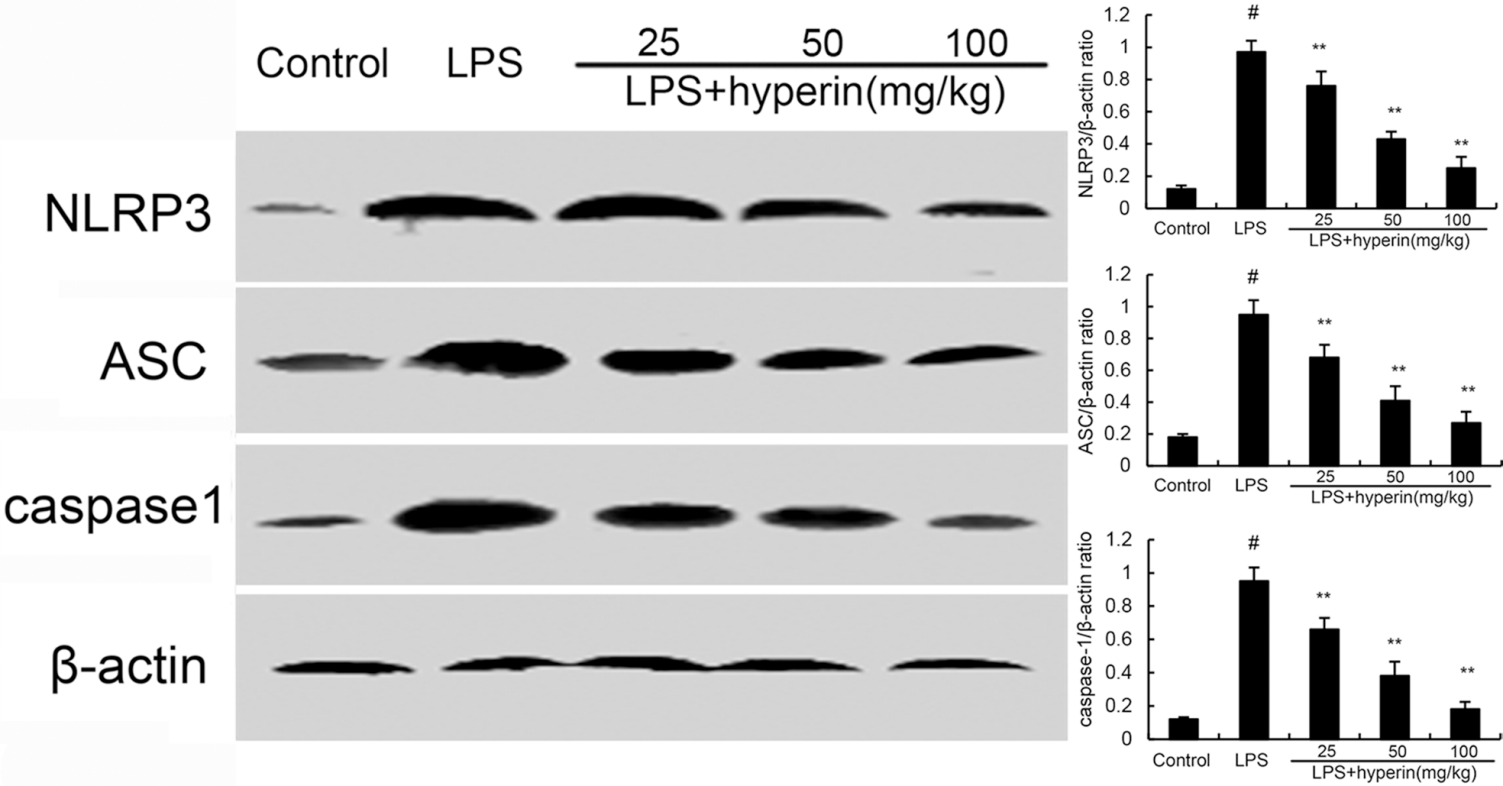
Hyperin inhibits LPS-induced NLRP3 activation The values presented are the means ± SEM (*n* = 12 in each group). ^#^*p* < 0.01 vs. control group, ^*^p < 0.05 and ^**^p < 0.01 vs. LPS group.

To further investigate the anti-inflammatory mechanism of hyperin, we examined the effects of hyperin on NLRP3 signaling pathway. As shown in Figure 5, compared with the control group, the expression of NLRP3, ASC, and caspase-1 were found to be dramatically increased in the LPS group. However, hyperin significantly suppressed LPS-induced NLRP3, ASC, and caspase-1 expression.

## DISCUSSION

In the present study, we evaluated the protective effects of hyperin on LPS-induced acute kidney injury in mice. The results showed that hyperin inhibited LPS-induced AKI by suppressing the levels of BUN and creatinine, as well as the production of TNF-α, IL-6, and IL-1β. Hyperin protected against LPS-induced AKI by inhibition of TLR4 and NLRP3 signaling pathways.

In this study, an animal model of AKI was established by LPS in mice. In this model, LPS significantly up-regulates the production of inflammatory cytokines, such as TNF-α, IL-6, and IL-1β. These cytokines have been demonstrated to play critical roles in the pathogenesis of LPS-induced AKI [13]. Studies showed that TNF-α and IL-6 were closely related to the extensive tubular damage [8, 14]. IL-1β also plays an important role in the pathogenesis of kidney inflammation and tissue damage [15]. Previous studies showed that inhibition of these inflammatory cytokines could protect against LPS-induced AKI [16]. To investigate the anti-inflammatory effects of hyperin, the effects of hyperin on inflammatory cytokines production were detected in this study. The results showed that hyperin protected against LPS-induced AKI by inhibiting inflammatory cytokines production.

LPS acts via TLR4 signaling pathway, which subsequently induces NF-κB activation and release of inflammatory cytokines [17]. Previous studies showed that TLR4 played an important role in LPS-induced acute kidney injury [18, 19]. NF-κB is a transcriptional factor that regulates a variety of inflammatory gene expression [20]. Studies showed that NF-κB activation is closely associated with the development of kidney injury [21]. Activation of NF-κB has been observed in mice of LPS-induced AKI [22]. To investigate the anti-inflammatory mechanism of hyperin, the effects of hyperin on TLR4 signaling pathway was detected. The results showed that hyperin dose-dependently inhibited LPS-induced TLR4 expression and NF-κB activation. NLRP3, a multiprotein complex, has been reported to activate caspase-1, which leads to the secretion of IL-1β [23]. Studies showed that NLRP3 inflammasome promotes renal inflammation and NLRP3 inflammasome knockout mice are protected against kidney injury [24]. In this study, we observed that treatment of hyperin significantly inhibited NLRP3 activation.

In conclusion, the results of this study provided evidence that hyperin has protective effects against LPS-induced AKI. The mechanism may be through the inhibition of TLR4 and NLRP3 signaling pathways.

## MATERIALS AND METHODS

### Chemicals and reagents

Hyperin (purity > 98%) was purchased from Preferred (Chengdu, China). LPS (Escherichia coli 055:B5) and Dimethyl sulfoxide (DMSO) was provided from Sigma Chemical Co. (St.Louis, MO, USA). Mouse TNF-α, IL-6 and IL-1β ELISA kits were purchased from Abcam (Cambridge, UK). Mouse NLRP3, ASC, caspase-1, TLR4 were purchased from Santa Cruz Biotechnology (Santa Cruz, CA). NF-κB p65, NF-κB p-p65, IκB, p-IκB, and β-actin were provided from Cell Signaling Technology Inc. (Beverly, MA). All other chemicals were of reagent grade.

### Animals and grouping

Sixty mice (BABL/c mice, 6–8 weeks) in this study were provided by center of Experimental Animals of Baiqiuen Medical College of Shandong University and all the experiments complied with Institutional Animal Care and Use Committee of Shandong University. For the model of AKI, mice were given with 15 mg/kg body weight of LPS in 50μl PBS via intraperitoneal injection. 24 h after LPS challenge, the mice were euthanized and the blood and kidney tissues were collected. Sixty mice were randomly separated into five groups, and each group contained 12 mice. Group 1 was treated as negative control, which received equal amount of PBS. Group 2 was served as LPS group, which received with 15 mg/kg body weight of LPS in 50μl PBS via intraperitoneal injection. Group 3–5 were served as hyperin (25, 50, 100 mg/kg) + LPS groups, which received 25, 50, 100mg/kg 1 h before LPS treatment.

### H&E staining of kidney tissues

Kidney tissues were collected and fixed in PBS containing 10% formalin. Then the fixed tissues were trimmed in adapted cube and embedded in paraffin. The paraffin sections (slice thickness is 5 microns) were stained with H&E staining. Finally, the sections were examined by microscope.

### Measurement of BUN and creatinine

After 24 hours of the treatment, the blood of each mouse was collected. In this study, AutoAnalyzer was employed to explore the levels of BUN and creatinine. The protocol was conformed to the instruction of the instrument.

### ELISA assay

Kidney tissues from different groupwere homogenized with PBS on ice, and then centrifuged at 4°C for 40 min at 12,000 rpm. Subsequently, the supernatants were collected to determine the expression of TNF-α, IL-6, and IL-1β. The levels of inflammatory cytokines were measured by using ELISA kits (R&D) according to the manufacturer's instructions.

### Western blot analysis

Kidney tissues were homogenized in protein extraction buffer and centrifuged at 10000 g for 10 min. The supernatants were measured using a BCA protein assay kit to obtain protein concentration. The proteins were separated on 10% SDS-PAGE and transferred to PVDF membranes. After being blocked with 5% fat-free dry milk, the membranes were incubated with the primary antibody at 4°C for 24 h. After washing three times with TBS/Tween20, the membranes were probed with HRP-conjugated secondary antibodies at room temperature for 1 h. Finally, the membranes were visualized with ECL-chemiluminescent kit (ECL-plus, Thermo Scientific, USA).

### Statistical analysis

The data are presented as the mean ± SEM. Statistical evaluation of the results were analyzed using one way ANOVA (Dunnett's *t*-test) and two-tailed Student′s *t*-test. Statistical significance was accepted at *P* < 0.05 or *P* < 0.01.
